# Molecular mechanisms of Sepsis attacking the immune system and solid organs

**DOI:** 10.3389/fmed.2024.1429370

**Published:** 2024-08-29

**Authors:** Zhaoyun Yang, Yan Gao, Lijing Zhao, Xuejiao Lv, Yanwei Du

**Affiliations:** ^1^Department of Respiratory and Critical Care Medicine, The Second Hospital of Jilin University, Changchun, China; ^2^Department of Rehabilitation, School of Nursing, Jilin University, Changchun, China; ^3^State Key Laboratory of Chemo/Biosensing and Chemometrics, College of Biomedical Sciences, College of Chemistry and Chemical Engineering, Hunan University, Changsha, China

**Keywords:** sepsis, pathogenesis, inflammation, apoptosis, pyroptosis, mechanism

## Abstract

Remarkable progress has been achieved in sepsis treatment in recent times, the mortality rate of sepsis has experienced a gradual decline as a result of the prompt administration of antibiotics, fluid resuscitation, and the implementation of various therapies aimed at supporting multiple organ functions. However, there is still significant mortality and room for improvement. The mortality rate for septic patients, 22.5%, is still unacceptably high, accounting for 19.7% of all global deaths. Therefore, it is crucial to thoroughly comprehend the pathogenesis of sepsis in order to enhance clinical diagnosis and treatment methods. Here, we summarized classic mechanisms of sepsis progression, activation of signal pathways, mitochondrial quality control, imbalance of pro-and anti- inflammation response, diseminated intravascular coagulation (DIC), cell death, presented the latest research findings for each mechanism and identify potential therapeutic targets within each mechanism.

## 1 Introduction

Sepsis was defined as a life-threatening organ dysfunction caused by a dysregulated host response to infection (1). This definition emphasizes the importance of both the dysregulated host’s responses to infection and the mechanism and severity of organ dysfunction due to infection (2–4). Approximately 48.9 million individuals worldwide receive a diagnosis of sepsis annually, resulting in 11 million fatalities, thus constituting 19.7% of the total number of global deaths (5).

Although there has been a decline in the mortality rate of sepsis, which can be attributed to the use of antibiotics, fluid resuscitation, and the implementation of various therapies aimed at supporting multiple organs (6, 7), more than 100 randomized controlled trials and research did not find out a single treatment that consistently saves lives in sepsis patients (6, 8, 9). It was believed that the reason may be a vast, multidimensional array of clinical and biologic features, which is called sepsis heterogeneity (6). Accordingly, the pathogenesis of sepsis remains complex (4), including activation of signal pathways, disorders of mitochondrial quality control, imbalance of pro-inflammatory and anti-inflammatory responses, dysregulation of cell death and so on. Hence, understanding the pathogenesis of sepsis is crucial for identifying novel therapeutic targets and developing effective therapies for intervention of sepsis. Here, we systemically reviewed relevant studies and focused on the molecular mechanism underlying the development of immune dysfunction and organ damage in sepsis.

## 2 Classic mechanisms of sepsis progression

### 2.1 Activation of signal pathways

**Figure 1:**
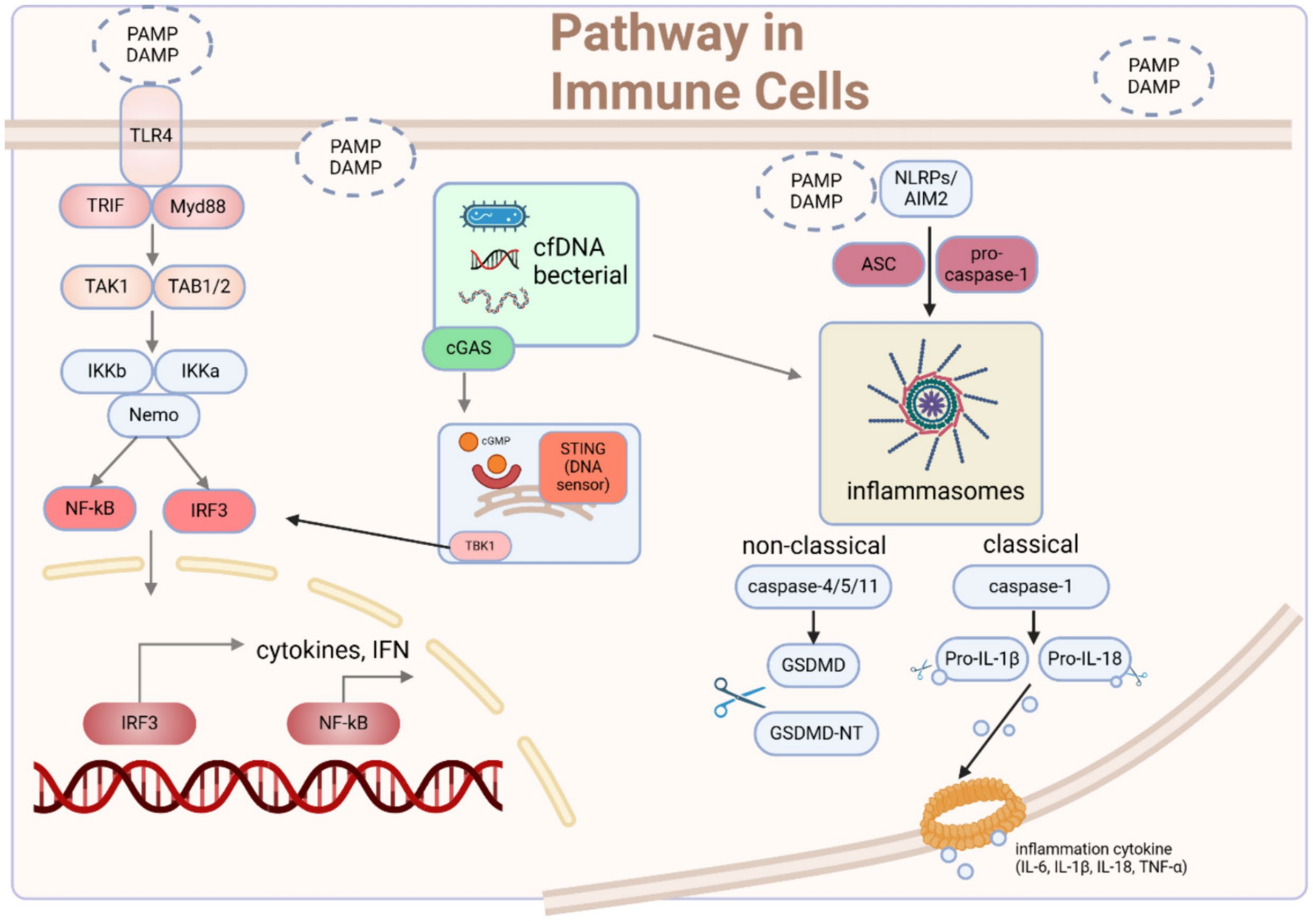
The signaling pathways through which immune cells recognize microbes and mediate immune responses. Upon initiation of sepsis, innate immune cells become activated upon recognition of DAMPs and PAMPs. Receptors located on the cell membrane and within the cell detect these danger signals, initiating various pathways that modulate the activation and regulation of innate immune responses. Typically, these pathways converge toward the IRF3 and NF-κB signaling pathways, which are crucial for the onset of early-phase inflammatory responses. Moreover, the activation of TLR4 by agonists serves as a vital priming signal for the initial steps of inflammasome activation, involving the upregulation of pro-inflammatory genes. Another important group of pathogenic substances and endogenous alarm signals are necessary to provide the second signal for the assembly of AIM2/NLRP3 inflammasomes, triggering the cleavage of caspases, GSDMD, and pro-IL-1β/18, leading to the canonical activation of inflammasomes and pyroptosis. Intracellularly, cfDNA originating from apoptotic cells or intracellular pathogens can be sensed by AIM2 and cGAS-STING, promoting inflammasome assembly and the phosphorylation of IRF3, ultimately inducing type I interferon responses and inflammasome activation. DAMP damage-associated molecular pattern, PAMP pathogen-associated molecular pattern, cfDNA cell-free DNA, ASC apoptosis-associated speck-like protein, GSDMD gasdermin D. Created with BioRender.com.

Sepsis, a complex and diverse syndrome, is distinguished from mild infection by its involvement of intricate signaling pathways and dysregulated host response, which contribute to its life-threatening nature (10) (Figure 1).

#### 2.1.1 The recognition role of PRRs situated at cell membrane or cytoplasm

The commencement of sepsis is triggered when the host cells identify pathogen-associated molecular patterns (PAMPs) or endogenous damage-associated molecular patterns (DAMPs) derived from microbes (10). Based on different modes of cell interference, pathogens are recognized by different pattern recognition receptors (PRRs). Specifically, pathogens on the cell membrane bind to TLRs, dectin1 and dectin2, while stimuli such as cell-free DNA (11) and mitochondrial DNA (12) that are engulfed by cells activate DNA sensors in the cytoplasm, such as Cyclic GMP-AMP synthase (cGAS). The interaction between cfDNA and cGAS leads to the generation of 2′3′-cGAMP, which serves as an activator for the stimulator of interferon genes (STING), ultimately leading to the secretion of interferons (IFNs) (11). Moreover, the activation of TLR4 also leads to the activation of the STING-IRF3 pathway (13). Consequently, the activation of STING results in the formation of homodimers, which are then facilitated to form oligomers which promote phosphorylation of downstream TANK-binding kinase 1 (TBK1) dimers. This phosphorylation event subsequently leads to the activation of the IRF3 and NF-κB signaling pathway (14, 15).

Numerous preclinical experiments have substantiated the crucial role of Toll-like receptors (TLRs), particularly TLR2 and TLR4, in mediating the pro-inflammatory effects of septic inflammation. These experiments, involving knocking down or inhibition of TLRs, provide substantial evidence for the significance of TLRs as pivotal PRRs. For example, in sepsis, TLR2^−/−^ mice exhibit enhanced survival rates, improved cardiac function, decreased production of cytokines in the blood and myocardial tissues (16), reduced depletion of immune cells (17), lower production of ROS and overall improved mitochondrial function (18). TLR4 serves as the primary identifier of sepsis induced by LPS, triggering downstream NF-κB and IFN pathways through the activation of MyD88 and TRIF. Treatment with LYRM03, an ubenimex derivative, can ameliorate the LPS-induced acute lung injury (ALI) in animals by attenuating Myd88-dependent LPS-TLR4 signaling pathway, including the p38 MAPK and IκB/NF-κB pathways (19). Losartan, soluble CD4, *Bifidobacterium bifidum* E3 with *Bifidobacterium longum* subsp. infantis E4 all exerts their effect in various organs to mitigate organ damage in sepsis, operating through the aforementioned pathways (20–23).

#### 2.1.2 NF-κB signaling

As one of the crucial signaling pathways that converges upon sepsis activation, the nuclear factor-κB (NF-κB) signaling pathway plays a crucial role in facilitating inflammatory responses across multiple organs during the progression of sepsis (24–26). In fact, genes encoding inflammatory mediators, such as TNF-α, IL-6, cyclooxygenase-2 (COX2), inducible nitric oxide synthase (iNOS), and adhesion molecules, have putative binding sites for NF-κB. These binding sites serve the purpose of activating gene expression (26). The crucial involvement of NF-κB in immune and inflammatory reactions is underscored, establishing it as a leading contender for selective deactivation. Macrophages, important innate immune cells, can be activated by bacterial components, such as LPS and acquire the M1 phenotype, then the notable elevation in TLR4 levels facilitates the activation of the NF-кB pathways, consequently leading to inflammation (21, 27). And that process could be inhibited by Ang-(1–7), which promotes macrophages toward M2 from M1 phenotype via ACE2-Ang-(1–7)-MAS axis (21). Furthermore, Artemisitene (ATT), a byproduct of the antimalarial medication artemisinin, could inhibit liver damage caused by LPS-induced ferroptosis by activating Nrf2/HO-1/GPX4 to against NF-κB (28). Similarly, the mutual competition among NF-κB and other pathways also explain the protective effect of Procyanidin B2 (PB2) on LPS-induced ALI, which activates PI3K/Akt pathway (29). Consequently, the inhibition of NF-κB activation has been recommended as a compelling therapeutic approach to mitigate harm to various organs and enhance survival rates of sepsis patients. What’s more, activation of alternative pathways to compete with the NF-κB pathway may also be a more optimal solution, as the NF-κB pathway is an integral component of the host’s normal defense mechanism (30).

#### 2.1.3 The JAK/STAT signaling

The Janus kinase/signal transduction and transcription activator (JAK/STAT) is another prominent inflammatory signaling pathways that is commonly activated during the whole sepsis process, including systemic inflammatory response syndrome (SIRS) and compensatory anti-inflammatory response syndrome (CARS) (31, 32). Considering the intimate correlation between JAK/STAT and NF-κB signaling pathways and the observation that inhibitors addressing both pathways demonstrate comparable anti-inflammatory properties (33), pharmaceutical agents possessing dual inhibitory capabilities might prove to be more efficacious in managing septic shock (31). C498-0670 (C498) from TargetMol Bioactive Compounds Library Plus (Cat. D7800), has shown great potential for further therapeutic applications by impeding the activation of STATs and p-IKKα/β in immortalized cell lines and primary peritoneal macrophages, in addition to reducing the expression of LPS-induced inflammatory mediators *in vitro* (31). Similarly, in rat model of sepsis, the administration of Tofacitinib demonstrates a significant reduction in acute lung injury and a notable improvement in survival rates through the inhibition of the JAK–STAT/NF-κB pathway (34). What’s more, the investigation into the correlation between miRNA and sepsis has emerged as a prominent area of interest in recent times. The role of miR-210 in sepsis-induced renal injury was elucidated that inhibiting miR-210 effectively suppresses the activation of the JAK/STAT pathway, leading to a reduction in both renal injury and inflammatory response in sepsis (35). And the acceleration of LPS-induced ALI is facilitated by the lncRNA MIR3142HG through the miR-95-5p/JAK2 axis (36). In conclusion, the potential significance of targeting JAKs/STATs as an approach to decrease mortality rates in patients with septic shock stems from their evident involvement in immunological dysfunctions and multiorgan failure. It is noteworthy that the abundance of animal studies conducted on JAK–STAT pathways and their widely recognized impacts on sepsis stand in stark contrast to the paucity of clinical trials in this area (32).

#### 2.1.4 The MAPK signaling

In sepsis, the mitogen-activated protein kinase (MAPK) pathway is consistently activated in conjunction with the NF-κB pathway, either independently or cooperatively stimulating the secretion of downstream inflammatory factors such as COX-2, TNF-α, IL-1β, IL-18, IL-6, and iNOS (23, 37, 38). Treatment with methanol extract of *S. crispa* (SCF4) significantly suppresses the LPS-stimulated TNF-α, IL- 6, and IL-1β production by attenuating the TLR4-related MAPK signaling in macrophages (39). Similarly, treatment with Ginsenoside Rb1 or fisetin (a natural flavonoid) mitigates the LPS-induced kidney damages by inhibiting IL-6, TNF-α, COX-2 expression, AKT activation and prevents from sepsis-mediated death in mice by down-regulating the MAPK signaling (40, 41). Treatment with Chinese medicinal herb Qiang Xin 1 (QX1) (42) inhibits microglia activation and pro-inflammatory cytokine production, and prevents cognitive dysfunction of septic mice by reducing the MAPK signaling. Furthermore, it is worth noting that despite research claiming the involvement of MAPK in regulating NF-κB in sepsis (41), the cited studies do not prove this point and have not thoroughly investigated the subordinate relationship between the two. They only demonstrate the enormous potential of simultaneously inhibiting MAPK and NF-κB in rescuing sepsis (43, 44). Further research is needed to explore the relationship between the two. In general, the NF-κB pathway remains an important potential therapeutic target for the early activation of the inflammatory response in sepsis, which may have significant implications for reducing the acute phase mortality of sepsis.

## 3 Disorders of mitochondrial quality-control

**Figure 2:**
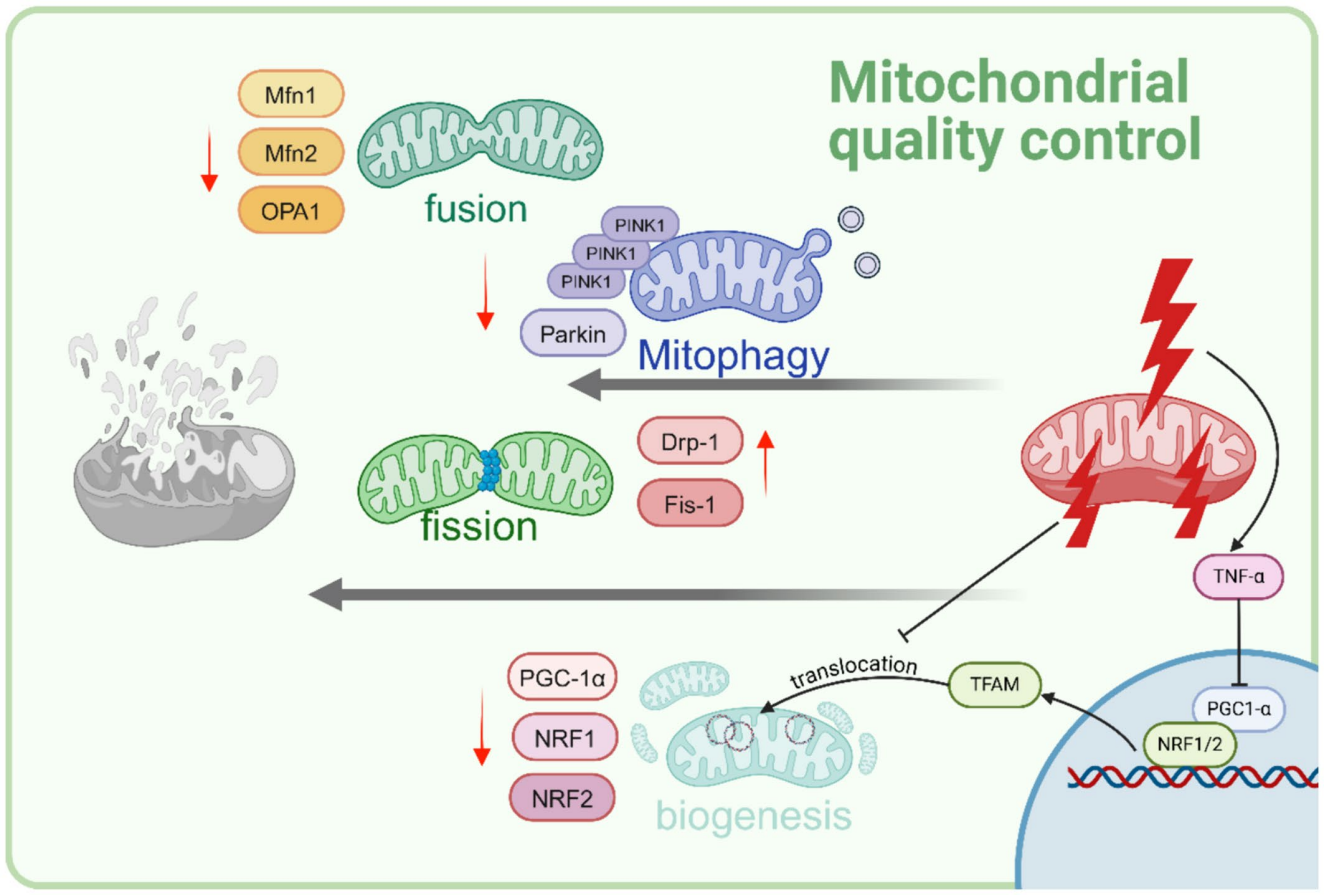
Sepsis disrupts cellular mitochondrial quality control. As sepsis advances, mitophagy is hindered. Furthermore, heightened mitochondrial fission and decreased mitochondrial fussion are evidenced as a reaction to sepsis-induced stress. There is a noticeable decrease in mitochondrial biogenesis is observed. This decline is predominantly influenced by increased levels of the cytokine TNFα, resulting in reduced levels of PGC-1α. Created with BioRender.com.

During sepsis, various mechanisms related to the maintenance of mitochondrial quality such as mitochondrial biogenesis, dynamics, and mitophagy are activated (45). Sepsis-induced exacerbation of organ malfunction is due to impaired regulation of mitochondrial quality control mechanisms, while the improvement in organ function also results from restoration of these regulatory mechanisms (46–50). What’s more, significantly, the dysfunction of mitochondria plays a crucial role in impairing the efficiency of the immune system. The presence of dysfunctional mitochondria contributes to the emergence of an excessively inflammatory state, thereby leading to unfavorable clinical consequences (51) (Figure 2).

### 3.1 Mitochondrial biogenesis

The regulation of mitochondrial biogenesis is governed by various molecular cues that are triggered by the need for energy. This process plays a significant role in augmenting the overall mitochondrial quantity and facilitating the restoration of the intricate mitochondrial network (45). The onset of mitochondrial biogenesis transcription factors occurs at an earlier stage in individuals who survive from sepsis, as opposed to those who do not survive (45, 47). This suggests that the prompt restoration of mitochondrial function holds significant importance in the management of sepsis. The central of mitochondrial biogenesis is TFAM-PGC1α-NRF1/2 pathway, in particular, TFAM is vital in the generation of new mitochondria (52). The diminished levels of intramitochondrial TFAM in individuals with sepsis, result in a concomitant reduction in the copy numbers of mtDNA, expression of mtND1, and overall cellular ATP content (53). The PGC-1α, play a crucial role in the replication of mtDNA. Recent studies have highlighted the significance of PGC-1α as the primary controller of mitochondrial biogenesis (54). In brain, PGC-1α, critical regulators of mitochondrial biogenesis, significantly decreased in CLP-induced sepsis mice, while the inhibitor of Fgr kinase reversed mitochondrial damage by activating the SIRT1/PGC-1α pathway (55). In the kidney, pectolinarigenin has been proved to restored the expression of PGC-1α, Opa1 in CLP-treated and reduced mitochondrial fragmentation (56). What’s more, restoration of the expression of the PGC-1α seems to be a crucial requirement for the recovery from LPS-induced acute kidney injury (AKI) (57). On the contrary, more severe consequence were observed in tubule-specific PGC-1α knockout sepsis mice (57). The administration of LPS in rats induces an increase in mitochondrial biogenesis. This effect is accompanied by the upregulation of PGC-1α and TFAM (58). Contrary to human discoveries, sepsis-induced mitochondrial dysfunction is marked by TFAM’s compromised translocation (59). However, the enhanced biogenesis observed in rats discussed above does not correspond to an enhancement in mitochondrial functionality. As mentioned above, the implementation of pharmacological substances to stimulate the initiation of mitochondrial biogenesis has been identified as a promising therapeutic approach against sepsis (45, 60). The mechanism underlying may be attributed to the decrease of inflammatory cytokines including TNF-α, IL-1β, and IL-6 and reduction of ROS generation by PGC-1α activation (61). Further research is needed to validate the hypothesis that recovery from sepsis relies on timely activation of mitochondrial biogenesis and a balanced biogenesis response. While excessive biogenesis may lead to structural abnormalities and impaired mitochondrial functionality, studies have suggested its importance in the recovery process.

### 3.2 Mitochondrial dynamics

Mitochondrial dynamics, specifically fission and fusion, encompasses the alteration of the structural composition of existing mitochondria (51).

During sepsis, the occurrence of mitochondrial dysfunction leads to the initiation of mitochondrial fission and the inhibition of mitochondrial fusion, potentially facilitating the removal of damaged mitochondrial components through redistribution and mitophagy. However, this shift can also lead to heightened oxidative stress and cell death in sepsis. This process, in turn, encourages the development of impaired mitochondrial fragmentation, ultimately leading to the detrimental consequence of various organ failure (62). Mitochondrial fission is predominantly influenced by Drp1, a prominent cytosolic GTPase (63). Drp1 undergoes translocation from the cytosol to the outer mitochondrial membrane, where it forms oligomers and facilitates mitochondrial fission via a constricting loop (63, 64). An increasing number of studies have observed the involvement of Drp1 in mitochondrial damage. Suppression of Drp1 or inhibition of mitochondrial fission using the Mdivi-1 (inhibitor of Drp1) exhibited a notable reduction in mortality caused by CLP (65). Moreover, this intervention effectively mitigated dysfunction observed in various organs such as the heart, liver, kidney, vascular smooth muscle, and intestine (66). Similarly, the involvement of S100a8/a9 in the mechanisms underlying sepsis-induced cardiomyopathy (*SIC*) is evident, likely through its activation of TLR4-ERK1/2-Drp1-dependent mitochondrial fission and impairment. Inhibiting S100a8/a9 was a promising therapeutic approach to mitigate the development of *SIC* in sepsis patients (67). These beneficial effects were achieved by impeding mitochondrial fission and mitigating mitochondrial dysfunction. It is worth noting that Drp1 is recruited to the mitochondria by partner proteins Fis1, Mff, MiD49, and MiD51 (68), however, not all partner protein inhibition can be regarded as a therapeutic approach for the treatment of sepsis. For instance, *in vivo*, the inhibition of Drp1-Mff either through pharmacologically or genetically expedites the progression of the pathological manifestations linked to neurodegenerative disorders (69). Latest study revealed that the utilization of Drp1-Fis1 inhibitors, such as P110 and SC9, may prove to be pivotal in preserving the functionality of mitochondria by effectively restraining excessive fission (63). In sepsis-induced AKI, the inhibition of Drp1-Fis1 pathway by reducing lactate levels and Fis1 lactylation attenuate the damage (70).

In contrast to mitochondrial fission, another crucial component of mitochondrial dynamics during the process of sepsis is mitochondrial fusion, which holds significant implications for preserving mitochondrial function and reducing organ damage (71). The process of mitochondrial fusion is primarily regulated by two proteins, known as mitochondrial fusion protein (Mfn) 1 and 2, as well as optic atrophy protein 1 (OPA1) (72). The activation of OPA1 has been identified as a potential therapeutic approach for sepsis. Several studies focusing on different pathway on various organs have provided evidence supporting this notion. In kidney, the deacetylation of YME1L1 by Sirt3 facilitates the promotion of mitochondrial fusion mediated by OPA1 and restore AKI, as it effectively inhibits the processing of L-OPA1 (71). While The administration of mesenchymal stem cell-derived microvesicles (MMVs) has the potential to transport Mfn2 to the intestinal epithelial cells and effectively enhance the equilibrium of mitochondrial dynamics following sepsis, ultimately leading to the restoration of both mitochondrial function and the integrity of the intestinal barrier (73). In ALI, both *in vivo* and *in vitro*, Dexmedetomidine exhibited the ability to alleviate the adverse effects of sepsis. This beneficial effect was achieved by maintaining a state of mitochondrial dynamic equilibrium through the activation of the HIF-1a/HO-1 signaling pathway and upregulation the expressions of Mfn1, Mfn2, OPA1 (74).

### 3.3 Mitochondrial autophagy

Mitochondrial autophagy (mitophagy) is a crucial form of selective autophagy that can experience depolarization and impairment in response to various stimuli, such as reactive oxygen species (ROS), inadequate nutrient supply, hypoxia, and inflammatory factors. Mitochondria play a crucial role in coordinating the immune response during sepsis; however, it is important to acknowledge that they can also unintentionally exacerbate the detrimental effects. The impairment of mitophagy results in the excessive activation of inflammatory signaling pathways, which in turn disrupts the equilibrium of immune function (75). The results of whole blood mRNA sequencing in 392 patients revealed that the mitophagy level observed in sepsis patients admitted to the ICU was found to be lower compared to those admitted to the Emergency Room. Moreover, sepsis patients with a higher Sequential Organ Failure Assessment (SOFA) score generally exhibited a lower level of mitophagy (76). These findings imply that a heightened mitophagy level may serve as a potential indicator for a favorable prognosis in sepsis (76). Previous research conducted on the receptor-interacting serine/threonine-protein kinase 3 (RIPK3) and PINK1/PARK2 axis has revealed that these signaling pathways play a crucial role in regulating tubular mitophagy in the context of septic AKI (77, 78). At the same time, PINK1 and Parkin mediated mitophagy also plays a protective role in renal ischemia–reperfusion injury or acute kidney injury caused by sepsis (79). Latest study revealed compelling evidence that the auto- and paracrine IGFBP-7 signaling contributes to the perpetuation of sepsis-induced Inflammation-coupling tubular damage (ICTD) through a novel mechanism: the rewiring of mitophagy mediated by NIX/BNIP3 (80). In sepsis-induced acute lung injury, the absence of Nrf2 exacerbates impairments in ATP synthesis, fatty acid oxidation, and respiration. Notably, research demonstrates that damaged lung epithelial mitochondria upregulate Nrf2 expression, consequently promoting mitophagy to maintain mitochondria function (81). In final analysis, the hindrance of mitophagy is regarded as a means to reinstate the damage caused by sepsis.

According to contemporary understanding, it is posited that mitigating heightened mitochondrial fission, advancing fusion and mitophagy in the course of sepsis, and fostering timely biogenesis subsequent to sepsis could potentially mitigate organ malfunction and enhance sepsis outcomes.

## 4 Imbalance of immune homeostasis: pro-inflammatory and anti-inflammation responses

**Figure 3:**
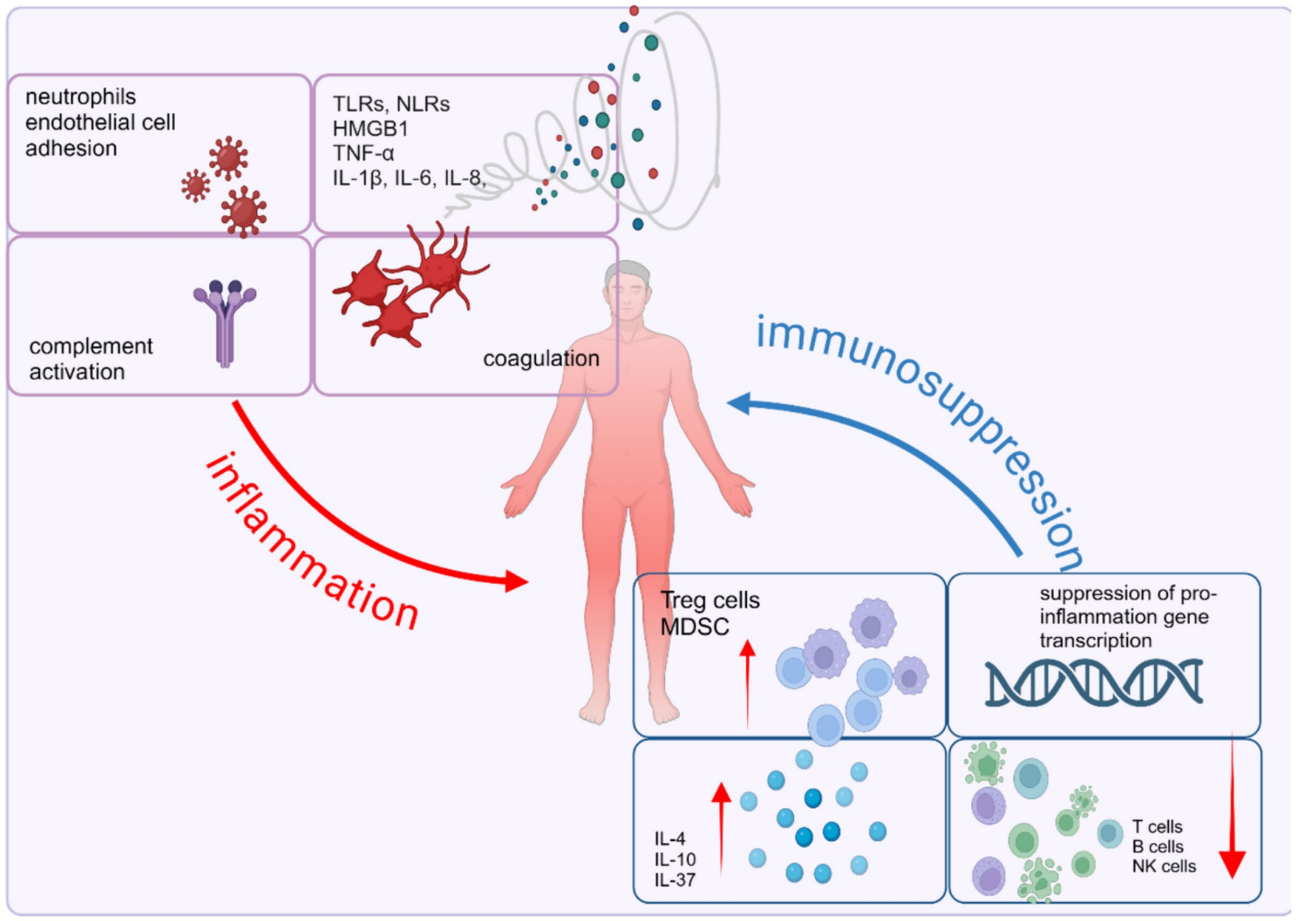
Imbalance of immune homeostasis in sepsis: pro-inflammatory and anti-inflammation responses. Sepsis is characterized by the simultaneous interplay of pro- and anti-inflammatory mechanisms. The proinflammatory response involves the release of pro-inflammatory mediators, activation of the complement and coagulation systems, and the release of alarmins due to necrotic cell death. On the other hand, the anti-inflammatory response is marked by impaired immune cell function caused by effector cell apoptosis, T cell exhaustion, heightened expression of suppressor cells, and the inhibition of pro-inflammatory gene transcription. Created with BioRender.com.

To commence this part, it is imperative to assert that there is no distinct division of sepsis into distinct pro-inflammatory and anti-inflammatory stages. Both gene expression data and results of clinical have supported the statement (82–87). Moreover, it has been demonstrated that the pro-inflammatory and anti-inflammatory reactions are concurrently controlled right from the initial stages of septic shock (88). The imbalance of pro-inflammatory and anti-inflammation responses lead to immunosuppression, characterized by a large amount of immune cell dysfunction and the activation of multiple signaling pathways (Figure 3).

### 4.1 Inflammatory cytokine storms

As reviewed above, several pathways are activated, which play a critical, mutual role in the process of sepsis. Subsequently, the level of various inflammatory cytokines rises remarkably, including high mobility group box-1 protein (HMGB1), TNF-α, IL-8, IL-6, and IL-1β (89–92), which impair host defense against pathogens, alongside their crucial involvement in the excessive systemic inflammation and resultant damage to organs during sepsis (93). Plenty of drugs, natural or artificial, were proved their protection effect on sepsis mouse models through inhibiting the activation of inflammatory cytokines via various signal pathways such as TLR4/NF-κB, MD-2/TLR4, ROS/NLRP3 pathways (94–96). However, the efficacy of drugs targeting TNF-α, IL-1β, or TLRs appears to be limited in *in intro* experiments. The drugs failed to enhance the survival rate of sepsis patients (7, 97–99). Recent research endeavors have redirected their attention toward the immunosuppressive phase of sepsis and innovative immunomodulatory therapies instead of targeting hyperinflammation (97).

### 4.2 Augmented secretion of anti-inflammatory cytokines

One of the mechanisms responsible for sepsis-induced immune suppression is the upregulation of the release of anti-inflammatory factors. The key anti-inflammatory cytokines associated with sepsis are IL-4, IL-10, and IL-37 (7). In sepsis, an elevation in the secretion of IL-4 occurs, which consequently transfers undifferentiated T cells into Th2 cells (100, 101). But latest research supported the positive role of IL-4 in training immunity which featured commonly associated with trained immunity, such as epigenetic reprogramming, elevated metabolic functioning, and modified transcriptomic reactions (102). And apoA1–I (103) L4, a fusion protein of apolipoprotein A1 (apoA1) and IL4, was developed to resolve sepsis-induced immune-paralysis (102). What’s more, Th2 cells, along with monocytes/macrophages, secreted IL-10, which inhibits the expression of TNF-α in monocytes, promote the proliferation of MDSCs and aggravate immunosuppression in advanced sepsis mice and patients (104, 105). In macrophage, many pathways participate in IL-10 production, including MSK/CREB, PI3K/Akt and TPL-2/ERK pathway. Recently, studies exhibited that IFN-β could promote IL-10 expression in macrophages and TRIM24 inhibited IFNβ/IL-10 axis signal during macrophage activation (106, 107). Similarly, it is reported that the upregulation of IL-37 in sepsis patients is of great significance as it has the potential to impede the proliferation and release of pro-inflammatory cytokines. This upregulation of IL-37 is closely correlated with the severity of immunosuppression induced by sepsis (7, 108).

### 4.3 The depletion of effective immune cells

In sepsis, there is a noticeable occurrence of apoptosis in immune cells including CD4 T cells, CD8 T cells, B cells, natural killer (NK) cells, and follicular dendritic cells (109). The postmortem studies of patients who died of sepsis revealed diminished numbers of splenic T cells in contrast to individuals who died from causes unrelated to infection (110). Exhaustion is apparent in CD8^+^ T cell reactions through the display of weakened cell division, compromised ability to destroy cells, and reduced generation of IL-2 and IFN-γ (110–112). Meanwhile, monocytes and macrophages have important roles in sepsis-induced immunosuppression (113). Notably, the expression of human leukocyte antigen–DR isotype (HLA-DR), a cell surface receptor used for antigen presentation in monocytes and macrophages diminish in sepsis, which correlate with impaired outcomes, including a higher incidence of nosocomial infections and increased mortality (114, 115). In septic patients, an upregulation of caspase-1 expression in various immune cells is observed when contrasted with its levels in individuals without underlying health conditions. The correlation between the occurrence of sepsis and the release of IL-18 as well as the rate of monocyte pyroptosis has been established (116). Autophagy and ferroptosis, alongside apoptosis and pyroptosis, are recognized as significant contributors to the development of sepsis-induced immunosuppression (117). Inhibition of autophagy has been shown to augment the antimicrobial efficacy of macrophages. Moreover, the suppression of autophagy has been observed to mitigate cytokine storms and vascular permeability (118, 119). Emerging evidence implicates ferroptosis in sepsis, as it directly exacerbates or promotes organ damage associated with this condition (120). Ferroptosis mediated by solute carrier family 39 member 8 (SLC39A8) plays a significant role in the depletion of monocytes among individuals with sepsis, thereby leading to immune system suppression. Conversely, the suppression of SLC39A8 has the potential to diminish lipid peroxidation induced by LPS (121).Another notable characteristic of immune suppression involves the reprogramming of monocytes and macrophages, resulting in a diminished ability to generate proinflammatory cytokines when exposed to bacterial agonists in laboratory conditions (109). Notably, using cross-species, single-cell RNA sequencing (scRNA-seq) analysis, latest research revealed the presence of a conserved subset of monocytes that exhibits high expression of S100A family genes and low expression of HLA-DR. These monocytes are predominantly found in late sepsis and are associated with an immunosuppressive response (122).

### 4.4 Excessive activation of regulatory cells

In addition of dysfunction of effective immune cells, the excessive activation of regulatory cells, such as Tregs and MDSC also contributes to sepsis-induced immunosuppression (7). There is an observed increase in the population of circulating Treg cells compared to other effective cells. This phenomenon has been documented in both animal and human studies, both in the circulation and in spleen tissue (123–125). The shift in lymphocyte population toward Treg cells, which have extensive regulatory and suppressive impacts on other immune cells, can be elucidated by their ability to resist apoptosis due to heightened expression of the antiapoptotic protein BCL-2 (111). The investigation into the function of Tregs in the immunosuppressive state induced by sepsis holds promise as a prospective avenue for further scholarly inquiry. In experimental models of sepsis, the population of MDSC is massively expanded (126). MDSCs exhibit immunosuppressive effects by employing diverse mechanisms including the degradation of L-arginine, the generation of ROS and RNS, the release of immunosuppressive cytokines such as IL-10 and TGF-β, and the induction of Tregs (127).

### 4.5 Disorder in cholinergic anti-inflammatory pathway

The cholinergic anti-inflammatory pathway (CAP) is a neuro-immunomodulatory pathway that operates by facilitating the release of acetylcholine (ACh) through the intricate interaction between the vagus nerve and the α7 nicotinic acetylcholine receptor (α7nAchR) (128). After Ulloa and Tracey demonstrated that nicotine enhances mouse survival in LPS and CLP-induced sepsis through the inhibition of HMGB1 release and the blockade of the NF-kB pathway by activating α7nAChRs, the stimulation α7nAChRs in combating sepsis became a novel potential therapeutic approach for sepsis (128, 129). Even 24 h after CLP-induced polymicrobial sepsis, the administration of choline continued to enhance the survival of mice, underscoring the significant impact of CAP in combating sepsis (130). Furthermore, the mechanism is closely associated with the functions of splenocytes (128). Researchs have demonstrated that the activation of α7nAChR leads to the suppression of TLR4 and CD14 expression via α7nAChR/PI3K signaling pathway (131, 132). A novel diarylheptanoid known as compound 28 holds promise as a potential therapeutic candidate for sepsis treatment by exhibiting agonistic properties toward the α7 nAchR-JAK2-STAT3 signaling pathway (133). What’s more, the general consensus is that the inhibition of pro-inflammatory cytokine release in macrophages leads to the generation of an anti-inflammatory response mediated by α7nAChR. The administration of GTS-21 (134) or PNU-282987 (135) demonstrated notable efficacy in reducing the population of M1-polarized macrophages and concurrently augmenting the abundance of M2-polarized macrophages within the pulmonary region following an LPS-induced ALI in mice. Consistent with this finding, ACh demonstrated an inhibition on LPS-induced elevation of IL-1β and IL-6, which is associated with the M1 phenotype and promotion on the production of IL-4 and IL-10, characteristic of the M2 phenotype (136). Notwithstanding, clinical trials examining the effects of α7nAChR agonist on the immune response in experimental human endotoxemia did not demonstrate any significant modulation (137). The establishment of the role of the neuro-immune regulatory reflex in sepsis-induced immunosuppression is yet to be determined in scholarly research. Further clinical and preclinical experiments are required to further elucidate the mechanism behind the varying effects of CAP in sepsis, in different species.

### 4.6 Metabolic reprogramming in immune cells during sepsis

In sepsis, macrophages, as innate immune cells, play a pivotal role in promptly responding to systemic infections, with their activation being crucial in various organ damages. In the context of LPS exposure, macrophages undergo a metabolic reprogramming from oxidative phosphorylation to glycolysis, resulting in elevated succinate concentrations and eliciting an inflammatory reaction which is called the differentiation toward the M1 phenotype. This transition is predominantly characterized by activation of succinate dehydrogenase (SHD), which have been confirmed in sepsis-ALI (138). In septic cardiomyopathy, M1 macrophages exhibit an increased expression of HIF-1α protein, participating in the enhancement of glycolysis. Recent studies have unveiled a novel compound capable of downregulating HIF-1α expression by deactivating SDH, thus inhibiting macrophage glycolysis and alleviating cardiomyopathy (139). Furthermore, lipid metabolism serves as another crucial pathway in regulating the immune function of macrophages. The stimulation of macrophages by LPS leads to triglyceride accumulation through the augmentation of fatty acid and glucose uptake, facilitation of glucose incorporation into lipids, elevation of triglyceride synthesis, and inhibition of triglyceride breakdown (138). It is noteworthy that citrate, upon exiting the mitochondria and entering the cytoplasm, leads to the production of fatty acids. The key enzyme in this process, ATP citrate lyase (ACLY), may play a significant role in enhancing lipid synthesis. In the context of sepsis-induced liver injury, the upregulation of TREM2 expression in macrophages is associated with an amelioration of hepatic lipid metabolism, a decrease in TG levels, and a mitigation of liver and lung injuries (140).

### 4.7 Polarization alterations in monocyte–macrophage

Macrophages play a crucial role in both innate and adaptive immunity, exhibiting a notable degree of heterogeneity and adaptability. Their ability to transition into various phenotypes with distinct functions is influenced by alterations in the local microenvironment of particular tissues, thereby contributing to immune regulation. An excess of pro-inflammatory mediators is released by M1 macrophages, whereas M2 macrophages predominantly secrete anti-inflammatory mediators. The dysregulation in the proportion of M1-like to M2-like macrophages is implicated in the pathogenesis of sepsis, leading to its onset and progression.Tong et al. found that MMP-9 induces macrophage polarization to the M1 phenotype through NF-κB pathway activation (141). Similarly, proteins such as SENP3, ERK, Notch1 are activated in sepsis, collectively activating the NF-kB pathway, promoting macrophage polarization toward the M1 phenotype (44, 142, 143). Tang et al. discovered that the induction of M2-like macrophage polarization in septic mice through *Schistosoma japonicum* infection can inhibit M1-like macrophage polarization, thereby reducing inflammatory mediators and enhancing survival outcomes, as evidenced by previous research findings (144). The polarization of macrophages from M1 to M2 in sepsis is considered a primary approach for treating sepsis through epigenetic modifications. Indeed, this approach has effectively alleviated mortality rates and organ damage in animal models of early sepsis (145). The E3 ubiquitin ligase ITCH serves as a suppressor of inflammation by negatively regulating the process. Diminishing its activity results in the ubiquitination of IL-1α, subsequently promoting the enhanced pro-inflammatory activation of macrophages (146). What’ s more, alteration of gene expression can impact the polarization status and functionality of macrophages through the control of non-coding RNA (147). And METTL3, a catalytic enzyme belonging to the methyltransferase-like 3 family, has been shown to play a pivotal role in promoting the polarization of M1 macrophages through the direct methylation of STAT1 mRNA (148).

Immunosuppressive treatments utilized in the management of sepsis encounter notable constraints, primarily due to the diverse nature of sepsis, leading to challenges in pinpointing and addressing specific immune responses. The precise timing for the implementation of such treatments remains ambiguous, posing risks of inefficacy or adverse effects when administered prematurely or belatedly. Future research on modulating the immune system of sepsis patients should focus on two main aspects. Firstly, identifying and validating biomarkers that accurately reflect the immune stages of sepsis patients. Secondly, determining the optimal timing for the administration of various potential drugs.

## 5 Diseminated intravascular coagulation triggered by sepsis

Disseminated intravascular coagulation (DIC) is a secondary complication that occurs in up to 80% of patients with sepsis (149). In approximately 35% of cases, it manifests itself overtly (150). DIC plays a crucial role in the development of multi-organ failure in sepsis, and its presence is closely linked to increased mortality rates. Despite is a significant contributor to organ injury in sepsis.

### 5.1 The significant release of TF

In the early stages, the predominant mechanism of DIC was recognized as the significant upregulation of tissue factor (TF) through inflammatory cytokines. TF is a transmembrane protein that, when combined with factor VIIa, triggers the commencement of blood coagulation. Coagulation in endotoxemia and sepsis models is predominantly influenced by the levels of TF present on macrophages and monocytes (10, 151). Furthermore, it was previously thought that activated cells, including endothelial cells, neutrophils and eosinophils, expressed TF. However, subsequent studies have revealed that these cells actually obtain TF from monocyte-derived microparticles via surface receptors (149). However, the process of forming these circulating soluble TF MPs has recently been discovered. Formation of GSDMD pores facilitated coagulation in viable macrophages by inducing calcium influx, leading to coagulation by promoting phosphatidylserine externalization. This process played a crucial role in triggering TF activation and facilitating the formation of cofactor-protease complexes within the coagulation cascade (152). In experimental sepsis models, inhibiting TF has shown to effectively prevent organ failure and reduce mortality rates. This not only helps to decrease coagulation but also plays a role in modulating the inflammatory response (153). Inhibiting DSDMD expression may represent an effective approach in the treatment of sepsis-associated DIC.

### 5.2 Endothelial dysfunction in Sepsis

Endothelial dysfunction plays a pivotal role in the development of sepsis, serving as a key factor in the onset of multi-organ failure through increased vascular permeability, activation of the coagulation system, facilitation of tissue edema, and impairment of vital organ perfusion. During sepsis, microbial substances have the ability to directly activate endothelial cells via PRRs (154), leading to subsequent activation of inflammatory pathways such as NF-κB and mitogen-activated protein kinases. Additionally, during this process, activated macrophage contribute to the reprogramming of endothelial cells toward a secretory phenotype through the generation of reactive oxygen species and cytokines (155). Angiopoietin-1, Angiopoietin-2, along with their tyrosine kinase receptors, Tie1 and Tie2, are pivotal in regulating vascular balance by engaging in diverse signaling pathways such as Akt and FOXO1. In sepsis, Ang2 functions as a biomarker indicating the severity of sepsis and is associated with the progression of the condition. In latest study, Endothelial-to-mesenchymal transition (EndMT) has likewise been identified involving in the development of sepsis induced by LPS, and IL-35 effectively alleviated endothelial dysfunction by mitigating EndMT. Moreover, IL-35 has the potential to reduce EndMT by blocking the NFκB signaling pathway (156). What’s more, Ivanka found that sepsis-induced DIC is facilitated by TRPM7 within ECs. The ion channel activity of TRPM7 and the functioning of α-kinase are crucial for the organ dysfunction resulting from DIC in sepsis, with their presence correlating with elevated mortality rates in septic conditions (157).

### 5.3 Platelets

**Figure 4:**
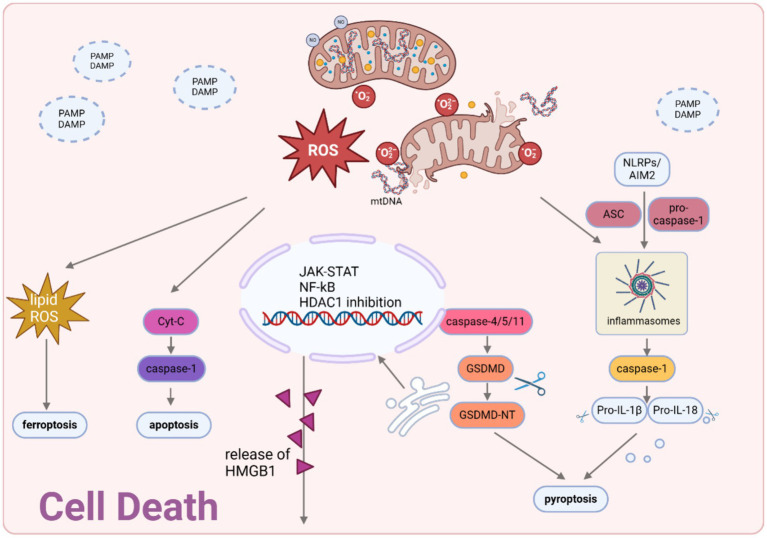
Cell death modalities in sepsis. Mitochondria-generated ROS and mtDNA act as triggers for the activation of the JAK–STAT pathway and the suppression of HDAC1, necessary for the hyperacetylation and movement of HMBG1 to the cytosol. Upon exposure to pathogenic stimuli, a significant amount of thiol-reduced HMGB1 is released through exosomes, functioning as inflammatory agents in sepsis. This redox state of HMGB1 interacts with AIM2 to initiate inflammasome activation and caspase-1-mediated responses, which are essential for inducing apoptosis/pyroptosis. Created with BioRender.com.

Recent studies have extensively investigated the function of platelets in the development of sepsis, highlighting their crucial role as connectors linking the hemostatic/coagulation system to the immune system (149). Platelets are typically among the initial cells to become activated in sepsis. The degree of thrombocytopenia is linked to the disease’s severity and prognosis. Platelets become activated through various stimuli such as DAMPs, inflammatory mediators, thrombin, and vWF. This activation leads to heightened levels of activated platelet P-selectin, thereby enhancing monocyte TF expression by interacting with the PSGL-1 receptors present on the surface of monocytes (3, 149), as well as α-granule and dense granule, is released from the plasma membrane to promote platelet cascade activation (158). Platelet aggregates, leading to the formation of small or large blood clots, occur through the activation of GPIIb/IIIa, facilitated by fibrinogen or vWF. NINJ1, a cell surface transmembrane protein, has been identified as the critical role in platelet activation and thrombosis in sepsis, which is closely to the platelet plasma membrane disruption (158). Furthermore, in addressing the limited efficacy of platelet transfusion therapy in sepsis (150), research indicates that platelet populations bearing CD40 ligands^hi^ produced by immune-skewed MKs from the spleen exhibit potent immunomodulatory functions, leading to a significant reduction in mortality in animal models (159) (Figure 4).

## 6 Cell death

### 6.1 Apoptosis

In sepsis, apoptosis is an inescapable pathophysiological outcome (160). Apoptosis occurs in both parenchymal and immune cells, causing multiple organ dysfunction and immune suppression.

Sepsis is associated with a strong depletion of CD4+ and CD8+ T cells, B cells and dendritic cells (DCs) as a result of apoptosis (110, 111, 160, 161). The induction of lymphocyte apoptosis in sepsis is believed to encompass both the extrinsic and intrinsic pathways (162). In the extrinsic pathway, the activation of caspase-8 is initiated by the Fas/Fas-ligand pathway, leading to the subsequent activation of caspase-3 and apoptotic program (7). Interestingly, the induction of immune cell apoptosis sepsis of mice is facilitated by FasL rather than by endotoxins or TNF-α (161). While in the intrinsic pathway, mitochondrial pathway, which is activated by BID, a pro-apoptotic member of the B-cell lymphoma-2 (Bcl-2) family protein, was responsible for the apoptosis. It has been discovered that there is a notable increase in the quantity of circulatory microvesicles (MVs), which contain an amount of caspase-1, being released from the monocytes of sepsis patients, inducing apoptosis in lymphocytes (163). Significantly, the enhancement of sepsis outcome in experimental models through the implementation of pharmacological or genetic approaches to impede lymphocyte apoptosis implies a direct correlation between lymphocyte loss and sepsis lethality (164).

Mitochondria-dependent parenchymal cell apoptosis plays a critical role in the pathogenesis of sepsis, causing microvascular dysfunction and organ failure. Cardiac dysfunction is an indisputable aspect of multiorgan failure with the participation of cardiomyocyte apoptosis (165, 166). As mentioned above, Bcl-2 and Bax play a pivotal role in governing the permeability of the mitochondrial membrane, as well as orchestrating the intricate process of cellular apoptosis by regulating the activation of cytochrome c/caspase-9/caspase-3 axis (167). Several researchers have raised a few pathways aiming to inhibiting Bcl-2-induced apoptosis. For instance, the activation of PI3K/Akt pathway restore apoptosis in heart, lung, and other vital tissues during LPS-induced sepsis (165, 168–170). Pharmacological approaches including compounds and Chinese traditional drugs also showed significant protection effect on sepsis organs by inhibiting cell apoptosis (165, 171–175). However, of note, sepsis-induced apoptosis involves both death receptor- and mitochondrial-mediated pathways, indicating the activation of multiple cell death stimuli in sepsis. Consequently, blocking a single apoptotic trigger is unlikely to prevent lymphocyte cell death in this disorder (111).

### 6.2 Pyroptosis

As the understanding of sepsis deepens, the concept of pyroptosis gradually emerges as a significant phenomenon in explaining the mechanisms underlying septic injury (176–179). In sepsis, controlled pyroptosis serves to impede the proliferation of intracellular pathogens, eliminate intracellular pathogens and impaired cells, and elicit an inflammatory reaction as a defense mechanism against infection. Nevertheless, an excessive manifestation of pyroptosis can instigate extensive cellular demise, resulting in septic shock, multiple organ dysfunction syndrome (MODS), or an elevated susceptibility to secondary infections (180–183). Pyroptosis is primarily triggered within innate immune cells. However, contemporary research has demonstrated the pyroptosis in nonimmune cells (180). During the sepsis, both classical and non-classical pyroptosis are involved. In the classical pyroptosis pathway, intracellular PRRs such as Nod-like receptor family pyrin domain containing 3 (NLRP3), NLR family caspase activation and recruitment domain (CARD), NLR containing 4 (NLRC4), and NLR family pyrin domain-containing 1B (NLRP1B) detect pathogenic stimuli and interact with pro-caspase-1 via the adaptor protein apoptosis-associated speck-like protein containing a CARD (ASC) (184–186). This interaction results in the formation of a multi-protein complex capable of activating caspase-1 protein. This process could be inhibited by *Cinnamomum cassia* (187), which inhibits the activation of NLRP3, NLRC4, and AIM2 inflammasomes, leading to a decrease in the secretion of IL-1β and an improvement in the survival rate of mice with septic shock induced by LPS. Similar mechanisms also exist in the protection effect of Glaucocalyxin A (188), ginsenoside metabolite protopanaxatriol (189) and myricetin (190). The activated caspase-1 plays a role in cleaving GSDMD and subsequently releasing N-GSDMD, which possesses the ability to form pores. These N-GSDMD oligomers then assemble within the cell membrane, leading to the formation of GSDMD pores. Consequently, the permeability of the cell membrane is altered, resulting in the ultimate cell lysis.

Of note, LPS trigger for the nonclassical pyroptosis pathway, functioning independently from the classical inflammasome (191). In the non-classical pyroptosis pathway, caspase-4/5 (human) and caspase-11 (murine) precursors are apical activators. Directly recognizing LPS and being activated by that, active caspase-4/5/11 cleave GSDMD to cause pyroptosis (192, 193). Caspase-11, in contrast to caspase-1, does not have the ability to directly cleave pro-IL-1β or pro-IL-18. Its mechanism involves the indirect activation of the NLRP3/ASC/caspase-1 pathway, leading to the maturation and release of IL-1β and IL-18 (194). Wang et al. discovered a compound called 8-ol, NSC84094, which effectively blocks HMGB1-mediated caspase-11 signaling. In animal experiments, 8-ol safeguard mice from sepsis (195). Natural alkaloid goitrin isolated from medicinal herbals, Artemisia argyi methanol extract also have been proved to be potential drugs for inhibiting caspase-11 activation, thus fighting against sepsis (196, 197). Some established drugs have been discovered to act as new medications for treating sepsis by influencing caspase-11, like heparin, Korean Red Ginseng (198, 199). This evidence suggests that inhibiting caspase-11-dependent non-canonical pyroptosis could be an effective approach in treating sepsis. Notably, the induction of intracellular caspase-11 also triggers the cleavage of pannexin-1, resulting in the efflux of K^+^, initiating the activation of the NLRP3 inflammasome and facilitate the activation of caspase-1. It shows that besides the classical pathway, the activation of caspase-1 can be induced through intercommunication with the caspase-11 pyroptotic pathway (200). Regardless of the means by which it is achieved, host-expressed members of the gasdermin family (GSDM) are the final effector proteins of pyroptosis. GSDMD is regarded as a novel and ideal target for drug development in sepsis (179). The findings of the study demonstrated that macrophages lacking GSDMD, when exposed to LPS and Gram-negative bacteria, do not lead to cellular scorching. Furthermore, it was observed that mice deficient in GSDMD exhibit an increased rate of survival following the induction of sepsis (192, 201).

### 6.3 Ferroptosis

Multiple research studies have emphasized ferroptosis’ involvement in sepsis and the resultant organ damage associated with it (202). Additional investigation is necessary as the intricate mechanisms and physiological significance of ferroptosis are not yet fully comprehended (203). Currently, the mechanisms underlying sepsis-organ damage caused by ferroptosis primarily revolve around the classical pathways of ferroptosis. In CLP-induced cerebrum, there is a growing occurrence of ferroptosis that supported by the observed decrease in GPX4 and SLC7A11 levels, elevation of ACSL4 and MDA levels, and the shrinkage of mitochondria (204–207). While research in septic cardiomyopathy (SCM) revealed that transmembrane protein 43 (TMEM43) protects against SCM by inhibiting ferroptosis in LPS-induced mice by downregulating the expression of P53, ferritin and increasing SLC7A11, GPX4 (208). Except of GPX4 pathway, in AKI, Zhang et al. provided evidence supporting the notion that overexpression of miR-124-3p.1 mitigates cellular injury by directly targeting the inhibition of LPCAT3-mediated ferroptosis, thus establishing miR-124-3p.1 as a potent inhibitor of this iron-dependent form of cell death (209). Inhibiting the process of ferroptosis presents itself as a compelling therapeutic approach in addressing the challenges posed by sepsis and the consequential organ damage that often follows. Indeed, a number of compounds have already exhibited promising therapeutic capabilities in addressing organ injuries associated with sepsis through the specific targeting of ferroptosis. Among them, acetaminophen (206), ferrostatin-1 (204, 207), and irisin (205) alleviate SAE by inhibiting ferroptosis. While Sodium hydrosulfide (NaHS) (210), melanin nanoparticles (211), ferrostatin-1 (212), and vitamin B6 (213) alleviate SCM by inhibiting ferroptosis. In-depth studies are currently underway on inhibitors targeting ALI and AKI, with the ultimate goal of inhibiting ferroptosis through the classical ferroptosis pathway. What’s more, ferroptosis of immune cells plays a crucial role in the immunosuppression in sepsis. T cell lipid peroxidation causes ferroptosis and weakens infection immunity. Gpx4 plays a vital role in maintaining the survival of CD8^+^ T cells and promoting the growth of CD4^+^ and CD8^+^ T cells during infection (214). In conclusion, the employment of pharmacologically targeted therapeutic medications, specifically those that hinder the process of ferroptosis, could potentially introduce a promising therapeutic approach in mitigating organ damage by sepsis.

## 7 Summary

Sepsis is a highly costly and severe medical condition to manage, and it is considered one of the most expensive pathological conditions. The estimated annual healthcare burden for septic shock is around $24 billion (7). Understanding the pathogenesis mechanisms of sepsis is crucial for the development of novel treatment approaches. Here, we summarized four classic mechanisms of sepsis progression, activation of signaling pathway, mitochondrial quality-control, imbalance of immune homeostasis, DIC, cell death and presented the latest research findings for each mechanism. Enhanced understanding of sepsis mechanisms is expected to facilitate the more precise and customized selection of treatments, leading to better outcomes in the future.
